# Early prediction of adverse outcomes in infants with acute bilirubin encephalopathy

**DOI:** 10.1002/acn3.51077

**Published:** 2020-06-04

**Authors:** Wenqing Kang, Xiao Yuan, Yaodong Zhang, Juan Song, Falin Xu, Dapeng Liu, Rui Li, Bangli Xu, Wen Li, Yanchao Cheng, Changlian Zhu

**Affiliations:** ^1^ Neonatal Intensive Care Unit, Zhengzhou Key Laboratory of Newborn Disease Research Children’s Hospital Affiliated to Zhengzhou University Zhengzhou 450018 China; ^2^ Henan Key Laboratory of Child Brain Injury Institute of Neuroscience and Third Affiliated Hospital Zhengzhou University Zhengzhou 450052 China; ^3^ Department of Neonatology Qilu Hospital of Shandong University Jinan China; ^4^ Center for Brain Repair and Rehabilitation Institute of Neuroscience and Physiology Sahlgrenska Academy Gothenburg University Gothenburg 40530 Sweden

## Abstract

**Objective:**

Acute bilirubin encephalopathy (ABE) remains one of the important causes of neonatal mortality and child disability, early identification, and intervention which could improve outcomes. The purpose of this study was to evaluate early predictors of adverse outcomes in infants with ABE.

**Methods:**

Newborns of gestational age ≥ 35 weeks and diagnosed with ABE were included in the study. Bilirubin‐induced neurological dysfunction (BIND) score, total serum bilirubin (TSB) peak value, and serum albumin levels were determined. Adverse outcomes were defined as death or survival with auditory dysfunction and/or cerebral palsy.

**Results:**

Eighty‐two infants were eligible for recruitment in the study. The outcome data from 76 ABE infants (92%) were used for analysis, of which 25 infants got adverse outcomes and 51 live a normal life. Univariate analysis for BIND score, TSB peak value, bilirubin–albumin ratio (B/A), albumin level, abnormal AABR, and neonatal sepsis was performed to elucidate the association with adverse outcomes. Bivariate logistic regression analysis showed B/A (OR 10.48, 95%CI: 1.55–70.81, *P *= 0.02) and BIND score (OR 3.68, 95%CI: 1.39–9.72, *P* = 0.01) were correlated with adverse outcomes. ROC curve analysis showed that B/A (≥8.9 mg/g), BIND score (≥6) could predict adverse outcomes of ABE separately; B/A in conjunction with BIND score could increase prediction sensitivity to 100%.

**Interpretation:**

Both B/A and BIND score can be used to predict adverse outcomes of ABE, and the combination of the two parameters can increase prediction sensitivity significantly.

## Introduction

Studies have revealed that increased levels of bilirubin is detrimental for nervous system, especially damage the basal ganglia, cerebellum, and brainstem, thus, resulting in acute bilirubin encephalopathy (ABE).^1^, ^2^ ABE in neonates is a significant cause of death or lifelong disability, including cerebral palsy (CP)^3^, ^4^ and auditory disorders.^5^ Incidence of ABE has decreased in the developed countries; however, ABE morbidity is still high in the developing countries. For example, in Nigeria, 159 cases of ABE were diagnosed in 1040 patients who were admitted for treatment of jaundice (15.3%).^6^ In China, 348 ABE cases were reported across 33 tertiary care referral centers, accounting for about 4.8% of the total number of newborns admitted in 2012.^7^

Clinical diagnosis of ABE and prognosis of associated adverse neurological outcomes are poor due to lack of objective laboratory examination standards. Furthermore, studies have shown that ABE should theoretically be completely preventable if detected and treated in time.^8^ Therefore, early monitoring and detection of bilirubin‐induced neurological dysfunction (BIND) is particularly critical.^9^ Previous studies demonstrated that total serum bilirubin (TSB) and bilirubin–albumin ratio (B/A) are closely related to the severity of ABE, and can serve as early predictors for adverse outcomes.^10^, ^11^ In this study, we focused not only on the above‐mentioned biochemical profile, but also on magnetic resonance imaging (MRI), automated auditory brainstem response (AABR), and clinical assessment such as BIND scores, to determine the early predictors of adverse outcomes in ABE.

## Subjects and Methods

### Subjects

Infants born at gestational age (GA) ≥35 weeks from January 2015 to December 2017 and diagnosed with ABE at Child’s Hospital, Third Affiliated Hospital of Zhengzhou University (Zhengzhou, China), and Qilu Hospital of Shandong University (Jinan, China) were enrolled in the study. All infants were followed up to 18 months of age, and neurodevelopment parameters were assessed. Risk factors of adverse outcomes were evaluated. ABE was defined as severe hyperbilirubinemia (TSB ≥ 340 *µ*mol/L, 17.1 *µ*mol/L = 1 mg/dL) with BIND.^12^ Neonates who presented with one of the following symptoms were excluded from the study: (1) Hypoxic‐ischemic encephalopathy, hypoglycemic encephalopathy, and neonatal encephalopathy with infection; (2) Congenital brain injury such as genetic, metabolic, and malformation diseases; (3) Congenital auditory deformity. As soon as they were diagnosed with ABE and admitted to the NICU, all infants received exchange transfusion, intensive phototherapy, and other treatment protocols, such as human immunoglobulin application and anti‐bacterial therapy, strictly in accordance with the guidelines of the American Academy of Pediatrics.^13^ Sepsis was identified based on clinical signs and symptoms as well as laboratory examination such as leukocytosis, leucopenia, and shift to the left of neutrophils, a positive C‐reactive protein screen regardless of whether blood culture was positive or not.^14^ Hemolysis was defined as hematocrit <35% with increased reticulocytes (> 6%) in patients positive for either direct/indirect Coombs test or release test.^15^ This study was approved by the ethics committee of Children’s Hospital Affiliated to Zhengzhou University and was carried out in accordance with the recommendations of Chinese Neonatal Society. All guardians/parents gave written informed consent in accordance with the Declaration of Helsinki.

### Study design and sample size

This study was designed as prospective nested case–control study, in which cases and controls are drawn from the ABE cohort based on the outcomes at the end points of 18 months after birth. The sample size was calculated based on BIND score. It has been reported that BIND score ≥ 4 had a specificity of 87.3% and a sensitivity of 97.4% for predicting adverse outcomes, with a percentage of 70% (P1) in infants with adverse outcomes and 30% (P0) in infants with normal outcome, then 24 cases in the adverse outcomes group and 48 cases in control group with normal outcome were need for a 1:2 case–control rate and a significance level of 5% with 90% power.^10^, ^16^

### Data collection

Detailed information about characteristics including weight at admission, sex, estimated gestational age, day of admission, days of ABE, and jaundice etiology (ABO hemolysis, Rh hemolysis, glucose 6 phosphate dehydrogenase deficiency, neonatal sepsis, and hemorrhage) was collected. TSB peak value and B/A ratio were measured and calculated in hospital. BIND score, which is used to assess mental status, muscle tone, and cry patterns, was also computed at admission. Cumulative BIND scores of 1–3, 4–6, and 7–9 were considered as mild, moderate, and severe ABE, respectively.^16^ An AABR instrument (ALGO 3i; Natus Medical Inc., Pleasanton, CA) was used to screen auditory function within one week after exchange transfusion. The results were recorded as “pass” or “refer for follow‐up” for each ear. Bilateral pass was considered “normal”. MRI was done within one week after exchange transfusion. Abnormal MRI was defined as bilaterally symmetrical hyperintensity in globus pallidi on T1‐weighted scans.

### Follow‐up

Surviving infants were followed up at 3, 6, 12, and 18 months by experienced neurologists, who were blinded to previous treatment. Diagnostic evaluation of auditory neuropathy was done by measuring the brain stem auditory evoked potential (MS‐230B, Nihon Kohden Corporation, Tokyo, Japan) at 3–5 months^5^ and was reported by a trained audiologist. Adverse outcomes were defined as death or survivors with auditory dysfunction and/or cerebral palsy.

### Statistical analysis

Data were analyzed using SPSS 21.0 software (IBM SPSS Statistics; IBM Corporation, Chicago, IL). Quantitative data with normal distribution were expressed as mean ± SD. Characteristics between adverse and normal outcome groups were compared using chi‐squared test. Risk factors for adverse outcomes were analyzed using bivariate logistic regression analysis. Receiver‐operator characteristic (ROC) curve and parallel test were used to evaluate the ability of different tests to predict adverse outcomes, and the cut‐off value was determined by maximum Youden’s index (*YI* = *Se* + *Sp* − 1). Delong test was used to compare area under the ROC curve (AUC). All statistical tests were two‐sided, and *P‐*values less than 0.05 were considered as a significant difference.

## Results

### Subject characteristics

**Figure 1:**
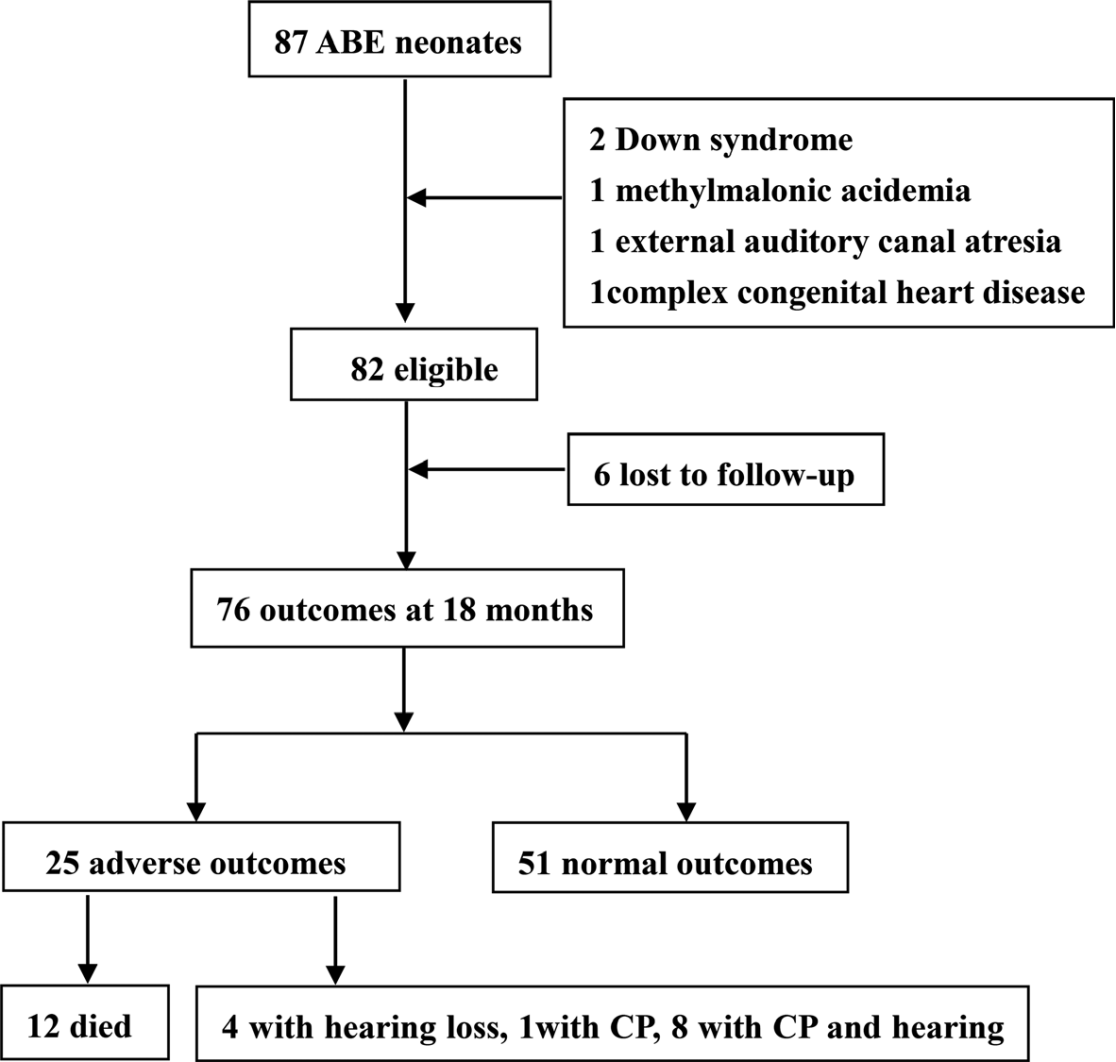
Study flow. The schematic flowchart depicting the subjects and the associated follow‐up outcomes. ABE, acute bilirubin encephalopathy; CP, cerebral palsy.

Eighty‐seven newborn infants with GA ≥ 35 weeks were diagnosed with ABE during the observation period in three NICU centers. Five cases were excluded on the basis of exclusion criteria. Of 82 cases that were eligible. Six cases were lost to follow‐up. The outcome data from 76 ABE cases (92%) including 51 normal outcomes and 25 adverse outcomes were used for analysis (Fig. 1). Twelve severe ABE infants died of ABE during hospitalization (4 with ABO hemolysis, 3 with Rh hemolysis, 4 with sepsis, 1 with ABO hemolysis and sepsis), and 13 infants turned out with poor outcome (4 cases with hearing disabilities, 1 with CP, and 8 with both hearing disability and CP). Thus, adverse outcome morbidity of 33% (25/76) and mortality of 16% (12/76) were observed in ABE cases.

Based on the follow‐up results, the infants were divided into adverse outcome (*n* = 25) and normal outcome (*n* = 51) groups. Univariate analysis showed significantly higher value of TSB (600.2 ± 109.4 *µ*mol/L vs. 463.4 ± 94.1 *µ*mol/L, *P* < 0.001), BIND score (6.6 ± 1.4 vs. 4.4 ± 1.7, *P* < 0.001), B/A (10.1 ± 1.7 mg/g vs. 7.5 ± 1.4 mg/g, *P* < 0.001), higher incidence of neonatal sepsis (36.0% vs. 11.8%, *P *= 0.01), lower serum albumin (33.8 ± 4.7 g/dL vs. 36.0 ± 3.9 g/dL, *P *= 0.04), and abnormal AABR (88.0% vs. 37.3%, *P* < 0.001) in infants with adverse outcome as compared to those with normal outcome. However, abnormal MRI, ABO/Rh hemolysis and hemorrhage had no association with adverse outcome (*P* > 0.05) (Table 1).

**Table 1:** Characteristics of ABE infants with different outcomes.

Variables	*n* (%)	Adverse outcome (*n* = 25)	Normal outcome (*n* = 51)	*P*‐value
Male, *n* (%)	48 (63.2)	17 (68.0)	31 (60.8)	0.54
Gestational age, w	38.5 ± 1.6	38.4 ± 1.6	38.5 ± 1.6	0.89
TSB (*µ*mol/L)	507.9 ± 118.0	600.2 ± 109.4	463.4 ± 94.1	0.00
ABO hemolysis, *n* (%)	31 (40.8)	11 (44.0)	20 (39.2)	0.69
Rh hemolysis, *n* (%)	8 (10.2)	3 (12.0)	5 (9.8)	0.76
Neonatal sepsis, *n* (%)	15 (19.7)	9 (36.0)	6 (11.8)	0.01
Hemorrhage, *n* (%)	4 (5.3)	1 (4.0)	3 (5.9)	0.22
MRI abnormal, *n* (%)	34 (54.8)	11 (73.3)	23 (48.9)	0.19
AABR abnormal, *n* (%)	41 (54.0)	22 (88.0)	19 (37.3)	0.00
Admission weight, g	3230 ± 509	3220 ± 504	3236 ± 517	0.90
Age of admission, d	5.6 ± 2.5	6.1 ± 2.8	5.3 ± 2.3	0.21
Age of ABE, d	4.6 ± 1.5	4.8 ± 1.9	4.5 ± 1.8	0.38
The interval time, d	1.0 ± 1.5	1.2 ± 1.5	0.8 ± 1.5	0.34
BIND score	5.1 ± 1.9	6.6 ± 1.4	4.4 ± 1.7	0.00
Albumin, g/dL	35.3 ± 4.3	33.8 ± 4.7	36.0 ± 3.9	0.04
B/A, mg/g	8.3 ± 1.9	10.1 ± 1.7	7.5 ± 1.4	0.00

TSB, total serum bilirubin; ALB, serum albumin; B/A, total serum bilirubin/serum albumin molar ratio; BIND score, bilirubin‐induced neurological dysfunction score. *P*‐value: compared variates between favorable outcomes with adverse outcomes. Quantitative data with normal distribution are presented as means ± SD. The interval time: time between the appearance of ABE symptoms and admission.

### Prediction of adverse outcomes

Variables with *P*‐value < 0.05 in univariate analysis, including BIND score, TSB peak value, B/A, albumin level, neonatal sepsis, and abnormal AABR (Table 1), were selected for bivariate logistic regression analysis. BIND score (OR = 3.68, 95%CI 1.39–9.72, *P *= 0.01) and B/A (OR = 10.48, 95%CI 1.55–70.81, *P *= 0.02) turned out to be correlated with adverse outcomes (Table 2).

**Table 2:** Bivariate logistic regression analysis of risk factors for adverse outcome of ABE.

Factor	*B*	SE	Wald	*P*	OR	95% CI
TSB	0.02	0.01	2.41	0.12	0.99	0.97–1.00
B/A	2.35	0.98	5.81	0.02	10.48	1.55–70.81
Albumin	0.54	0.51	1.15	0.28	1.72	0.64–4.63
BIND	1.30	0.49	6.93	0.01	3.68	1.39–9.72
AABR	0.29	1.37	0.05	0.83	1.34	0.09–19.68
Sepsis	0.36	1.04	0.12	0.73	1.43	0.19–11.03
Constant	5.19	8.65	0.36	0.55	0.01	

TSB, total serum bilirubin; B/A, total serum bilirubin/serum albumin molar ratio; BIND score, bilirubin‐induced neurological dysfunction score. *P*‐value: logistic analysis was made and *P* < 0.05 means significant difference.

**Figure 2:**
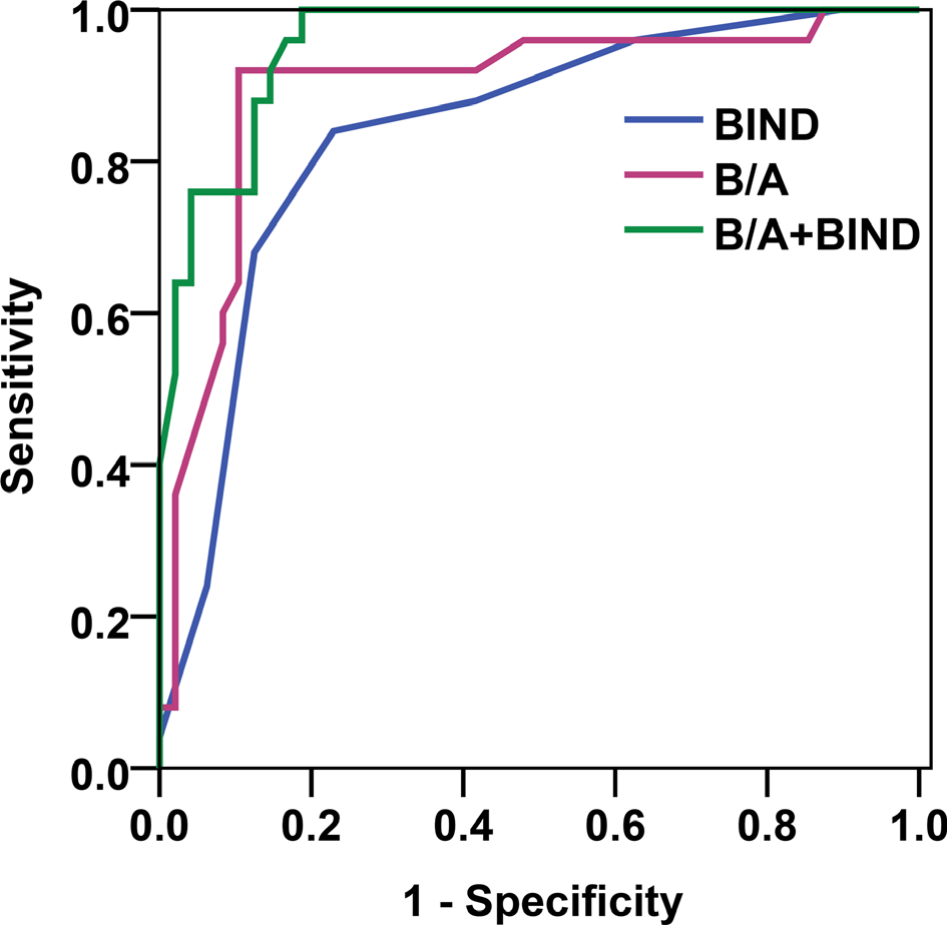
ROC curve of B/A, BIND score, and B/A in conjunction with BIND to predict adverse outcomes in ABE. Area under the ROC curve (AUC) for B/A was 0.895 and the cut‐off value was 8.9 mg/g. AUC for BIND score was 0.839, whereas the cut‐off value was 6. AUC for B/A in conjunction with BIND was 0.957 with a sensitivity of 100% and specificity of 81%. BIND, blue line; B/A, purple line; BIND combined B/A, green line.

ROC analysis revealed that B/A of 8.9 mg/g (AUC 0.895) can predict adverse outcome with a sensitivity of 92.0%, a specificity of 90.2%, a positive predictive value of 82.1%, and a negative predictive value of 95.8%. BIND score of 6 (AUC 0.839) with a sensitivity of 84.0%, a specificity of 77.1%, a positive predictive value of 65.9%, and a negative predictive value of 90.2%. AUC of B/A in conjunction with BIND score was 0.957 with a sensitivity of 100% and a specificity of 81% (Fig. 2), the combination increased sensitivity, but decreased specificity compared to B/A alone (Table 3). The accurate value of combined test was higher than that of B/A (*z* = 1.28, *P *= 0.01) or BIND (*z* = 2.23, *P *= 0.001) alone (Table 4).

**Table 3:** Predict values of B/A, BIND, and B/A in conjunction with BIND.

Variable	Sensitivity (%)	Specificity (%)	PPV (%)	NPV (%)	Youden’s index
B/A	92.0	90.2	82.1	95.8	0.82
BIND	84.0	77.1	65.6	90.2	0.61
B/A + BIND	100.0	81.3	84.8	100.0	0.81

PPV, positive predictive value; NPV, negative predictive value.

**Table 4:** Comparative analysis of AUC between B/A or BIND and B/A + BIND.

Variable	AUC	SE	95%CI	*z*	*P*
B/A	0.895	0.044	0.809–0.981	1.28^1^	0.01
BIND	0.839	0.049	0.742–0.936	2.23^1^	0.001
B/A + BIND	0.957	0.020	0.917–0.997		

^1^ Compared with B/A + BIND.

## Discussion

ABE remains one of the severe clinical problems that can cause neonatal death or neurological disability and is more important in the developing countries. Studies have shown that early diagnosis and efficient treatment could prevent neurological sequelae.^2^, ^8^ In this study, we demonstrated B/A value and BIND score are associated with adverse outcomes of ABE; both B/A and BIND can be used to predict adverse outcomes of ABE, and combination of B/A and BIND can increase prediction sensitivity.

Isoimmunization hemolytic disease is associated with increased incidence of severe hyperbilirubinemia in neonates.^17^, ^18^ In our study, 39 (51%) of 76 infants had ABO hemolysis (31 cases) and Rh hemolysis (8 cases), however, we did not observe any correlation between ABO hemolysis or Rh hemolysis and poor outcomes. This could be attributed to the targeted therapies (e.g., human immunoglobulin use) available nowadays,^19^ and timely intervention – the admission time of hemolysis infants (4.9 days) was earlier than the total average admission time (5.6 days). Even though there was no significant difference between the age of admission and age of onset of ABE, the interval between the appearance of ABE symptoms and admission was greater for infants with adverse outcomes (1.3 days) than that for infants with normal outcome (0.8 days). This means that delayed treatment may be a high‐risk factor for adverse outcomes of ABE.^20^ This also emphasizes the importance of recognizing the potential harm that jaundice can cause.^21^ Hemorrhage has also been considered as a risk factor for severe hyperbilirubinemia,^22^ however, no such relation was noted in this study. In this study, we observed nine of 15 septic infants (6 had positive blood cultures and negative CSF cultures) had poor outcomes, of which four died in neonatal period. It is well known that sepsis increases the total serum bilirubin levels as well as permeability of the blood–brain barrier, thus resulting in entry and accumulation of free unconjugated bilirubin in the CNS.^23^, ^24^ Consistent with this, we also found that sepsis increased the incidence of adverse outcomes in ABE infants.

TSB has been extensively used as an indicator of timely intervention of hyperbilirubinemia in clinics, but the accurate value of TSB to predict the occurrence and prognosis of ABE is controversial.^25^ In this study, we find no correlation between TSB and adverse outcomes. Notably, there was one infant with adverse outcome, and the highest TSB level was 398.3 *µ*mol/L, which may distort our findings, since there were only 25 infants with adverse outcomes in the final analytical. Moreover, in this study, 23 of 25 (92%) infants with poor outcomes got neonatal hemolysis or sepsis, which might also confound the TSB threshold for ABE.

Studies have confirmed that it is free unconjugated bilirubin not TSB causes CNS injury,^26^ but there was unavailable free unconjugated bilirubin detection method in most of the clinical practice.^27^ The B/A, including the two of three factors determining free bilirubin (TSB, albumin, and albumin‐binding affinity values), was thought to indirectly reflect the concentration of free bilirubin,^28^ and was concerned about whether it can be used to predict the occurrence and prognosis of ABE. It has been reported that a B/A of 8 mg/g can predict ABE with a sensitivity of 100% and specificity of 94%.^29^ Another cohort study showed that both TSB and B/A were strong predictors of neurotoxicity, but B/A did not improve prediction over TSB alone.^10^ In this study, a B/A of 8.9 mg/g (AUC 0.895) can predict adverse outcomes with a sensitivity of 92%, specificity of 90.2%, PPV of 82.1%, and NPV of 95.8%.

Next, we also noticed higher BIND scores (6.6 ± 1.4) in infants were associated with adverse outcomes. In this study, all infants with adverse outcome had BIND scores of 5–9, and the dead infants had BIND scores of 7–9. ROC analysis showed that BIND score ≥ 6 can predict adverse outcomes with a sensitivity of 84% and specificity of 77.1%, which is in agreement with previous studies.^16^, ^30^ Considering that BIND assessment is convenient to execute, it is worthwhile to extend its application in the clinics. However, although AUC of B/A (0.895) was slightly higher than that of BIND (0.839), the accurate value of B/A or BIND alone to predict adverse outcome of ABE were moderate. ROC of parallel tests analysis identified that B/A (≥ 8.9 mg/g) in conjunction with BIND score (≥6) with a sensitivity of 100% and specificity of 81% (AUC 0.957). The sensitivity was significant higher in the combination than that of B/A or BIND alone.

Furthermore, previous studies have indicated that myelination in neonates, which is also visualized as high T1‐signal because of its high lipid content, leading the predictive value of early MRI might be hindered by confounding abnormal T1‐signal with “normal” T1‐signal reflecting myelination.^31^ Consistent with this, we also found no predictive value of early MRI in adverse outcomes of ABE, which is in line with our previous study.^11^ Auditory system is highly sensitive to bilirubin neurotoxicity,^5^ and AABR has been an indicator for neurological sequelae of bilirubin toxicity.^5^ However, in this study, we found that AABR in neonates was not a good predictor of adverse outcomes. There were 19 (25%) infants with early AABR abnormality, but no hearing disability or CP with follow‐up, it indicates that part of neonatal abnormal AABR were temporarily or reversible. This could be that most of the infants, who were diagnosed with hemolysis, sepsis, or hemorrhage, had been treated successfully. Another reason could be that AABR being an electrophysiological response needs dynamic monitoring in order to obtain a systemic and comprehensive assessment.

The primary limitation of this study was our inability to explore more predictors, such as amplitude‐integrated electroencephalography, diffusion tensor imaging, and magnetic resonance spectroscopy, all of which have been tested as promising tools for assessment and prognosis of ABE.^32^ Another limitation is restricted etiology of ABE due to geographic differences in China. For example, G6PD deficiency is rather dominant in south China, whereas all the subjects in our study came from midland of China and no infants were diagnosed with G6PD deficiency. Lastly, 18 (24%) infants did not have clear causation of extreme hyperbilirubinemia resulting in typical ABE, future studies can be aimed to identify the mutations or polymorphisms in genes involved in bilirubin production or metabolism using next generation sequencing and elucidate the underlying causes of hyperbilirubinemia that lead to ABE.

In summary, this study with a fairly large number of ABE infants with a follow‐up to 18 months indicates that both clinical BIND score and B/A value can predict adverse outcomes in ABE infants. More gratified is the combination of the above two parameters significantly increases the prediction sensitivity. This study demonstrates that it is possible for early prediction of adverse outcomes for the infants with ABE, which would be more useful for the clinicians to take a more prompt and aggressive intervention on those with a high risk of adverse outcomes to reverse bilirubin‐induced neurological dysfunction during the early phases of ABE.

## Conflict of Interest

The authors declare that the research was conducted in the absence of any commercial or financial relationships that could be construed as a potential conflict of interest.

## Authors’ Contributions

CZ and WK designed the research. CZ, WK, XY, and YZ analyzed the data. WK, XY, JS, and CZ wrote the paper. WK, FX, and WL supervised clinical diagnosis, treatment, and evaluation; DL, RL, BX, and YC were responsible for recruitment, clinical evaluation, and treatment, as well as clinical data collection; XY, DL, RL, and BX were responsible for follow‐up and outcome evaluation.
